# Comparing the Knotless Tension Band and the Traditional Stainless Steel Wire Tension Band Fixation for Medial Malleolus Fractures: A Retrospective Clinical Study

**DOI:** 10.1155/2016/3201678

**Published:** 2016-05-12

**Authors:** Michael W. Downey, Kyle Duncan, Victor Kosmopoulos, Travis A. Motley, Brian B. Carpenter, Fadeke Ogunyankin, Alan Garrett

**Affiliations:** ^1^Foot & Ankle Division, Department of Orthopaedic Surgery, John Peter Smith Hospital, Fort Worth, TX 76104, USA; ^2^Department of Orthopaedic Surgery, Bone & Joint Institute, University of North Texas Health Science Center, Fort Worth, TX 76104, USA; ^3^Department of Materials Science and Engineering, University of North Texas, Denton, TX 76107, USA; ^4^Department of Orthopaedic Surgery, John Peter Smith Hospital, Fort Worth, TX 76104, USA

## Abstract

The traditional stainless steel wire tension band (WTB) has been popularized for small avulsion fractures at the medial malleolus. Despite the tension band principle creating a stable construct, complications continue to arise utilizing the traditional stainless steel WTB with patients experiencing hardware irritation at the tension band site and subsequent hardware removal. Coupled with hardware irritation is fatigue failure with the wire. The goal of this investigation was to retrospectively compare this traditional wire technique to an innovative knotless tension band (KTB) technique in order to decrease costly complications. A total of 107 patients were reviewed with a minimum follow-up of 1 year. Outcome measures include descriptive data, fracture classification, results through economic costs, and fixation results (including hardware status, healing status, pain status, and time to healing). The KTB group had a 13% lower true cost as compared to the WTB group while the fixation results were equivocal for the measured outcomes. Our results demonstrate that the innovative KTB is comparable to the traditional WTB while offering a lower true cost, an irritation free reduction all without the frustration of returning to the operating room for additional hardware removal, which averages approximately to $8,288.

## 1. Introduction

Fracture repair is a continuous evolving process that requires appropriate reduction and stabilization in preparation for bone healing through osteosynthesis. The AO definition of the tension band principle focuses on converting a tensile force into a compressive force at the opposite cortex while acting as a buttress [1]. The most common locations for tension band repair in the literature are olecranon fractures, patella fractures, and medial malleolar fractures with the idea that small avulsion fractures or fractures in close proximity to an articular surface have compressive reduction to allow for early motion and the best functional outcome [1].

Medial malleolus fractures have been described in the literature through variable mechanism of injuries, with both direct and indirect influence on the specific pattern. Transverse type fractures, with or without comminuted association, have been described with tension band fixation and typically start or end at the medial malleolus in association with an external rotational force with a pronatory or supinatory foot position. Fixation of these fracture patterns has been documented with either a traditional stainless steel wire tension band (WTB), 18 or 22 gauges, or a 2-parallel-screw technique.

The biomechanical principles behind the tension band technique of utilizing the compressive force as a traction mechanism date back over 100 years in architecture and engineering work with similar principles emphasized in orthopaedic plate and screw designs [1]. Multiple bench-top and clinical studies have demonstrated a similar, if not superior, construct with tension band wiring in comparison to screw/plate designs often referred to as tension band plating [1]. Though there are subtle differences in approach, they both have common goals of resisting displacement/movement, preventing nonunion through a stable construct, all while avoiding adjacent soft tissue necrosis/ischemia.

Despite the tension band principle creating a stable construct, complications continue to arise utilizing the traditional stainless steel WTB with 15% of patients experiencing hardware irritation and subsequent hardware removal [2, 3]. Coupled with hardware irritation is fatigue failure with the stainless steel wire, as a wire fails due to repeated off-axis bending loads [1]. Preventing repeated wire failure can be problematic and requires finesse intraoperatively. Aiming for a design with a simplified approach, a stable construct, allowing for fracture healing with less irritation and avoiding the potential for additional procedures, is beneficial.

A recent biomechanical study introduced a knotless tension band (KTB) technique for fixation of medial malleolar fractures and suggested that such a low-profile system may have the advantage of reducing hardware irritation and thus the need for additional procedures and costly returns to the operating room [4]. The study compared tension band construct stiffness and strength between an 18-gauge stainless steel WTB and a novel KTB technique. Results demonstrated the KTB construct to be 7.7% stronger and 33.2% stiffer while requiring a 36.7% greater force to displace the fracture by 2 mm. The current clinical study aims to retrospectively evaluate factors including cost (e.g., initial system costs and return to operating room costs for failure/revision/removal of hardware) and fixation results (e.g., hardware, healing, pain status, and time to healing) between the traditional stainless steel WTB and the novel KTB techniques.

## 2. Methods and Measures

To be included in the study, patients had to have undergone fixation of their ankle fracture with the Arthrex (Naples, Florida, USA) KTB system (surgical procedure detailed below) or with a traditional stainless steel WTB system, demonstrate compliance, and have a minimum 1-year follow-up. After the study was reviewed and approved by the institutional review board, the electronic medical record system was used to identify patients meeting the aforementioned criteria through the attending surgeon's practices (Alan Garrett, Travis A. Motley, Brian B. Carpenter). A retrospective chart assessment was performed on all patients that were operatively fixed with the two different tension band systems (WTB versus KTB) from 01 June 2008 to 31 December 2013. Exclusion criteria involved noncompliance, any previous ankle fractures that were previously fixated with plate or screws and resulted in a nonunion or malunion, patients less than 18 years of age, pregnant women, and prisoners.

**Figure 1:**
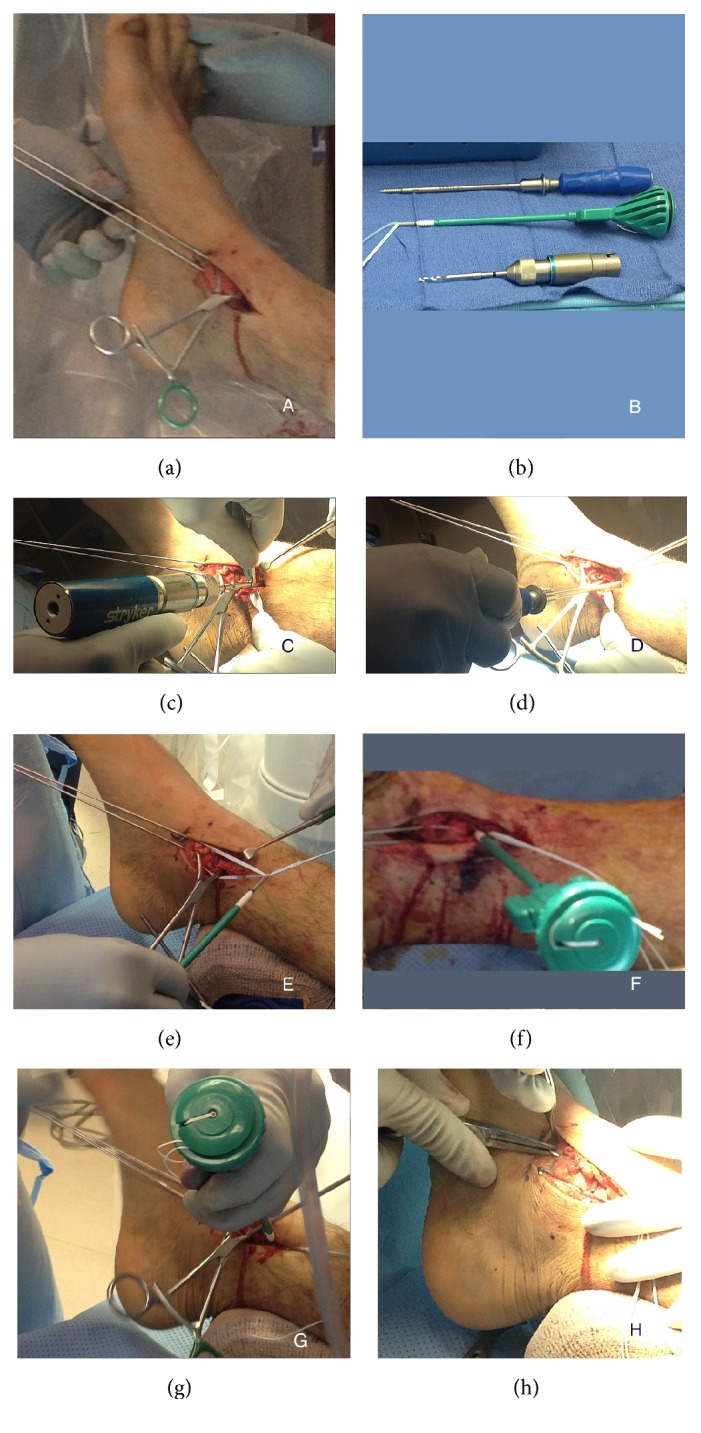
(a) demonstrates reduction of the fracture with two parallel 0.062 k-wires for the tension band effect; (b) shows the 3.4 mm drill, the Arthrex SwiveLock*™* tap, the Arthrex no. 2 FiberTape*™*, and Arthrex 4.75 mm SwiveLock anchor; (c) shows that the 3.4 mm drill was utilized for the anchor hole, being careful to maintain equidistance from the fracture site to the distal portion of the medial malleolus for the anchor hole; (d) shows that the 4.75 mm SwiveLock tap was utilized down to the laser line; (e) demonstrates the FiberTape being wrapped around the distal k-wires and both ends being placed through the 4.75 mm SwiveLock eyelet; (f) shows the FiberTape being tightened to appropriate tension and the SwiveLock anchor was advanced burying the anchor past the black line with the Arthrex AO handle; (g) shows the mallet being used to advance the anchor; (h) shows that the fracture reduced and in adequate anatomical alignment the k-wires were bent, cut, and rotated to avoid soft tissue irritation.

All KTB and WTB surgeries were performed within one institution (John Peter Smith Hospital, Fort Worth, Texas, USA). The KTB requires minor change in approach compared to the WTB and is detailed below. The WTB procedure is well established with details available in the literature [1] and thus omitted here. For the KTB technique, a curvilinear incision was utilized over the medial malleolus being careful to protect the saphenous nerve and vein; the incision was deepened in a layer-by-layer fashion until the fracture was visualized. The fracture site was cleaned of hematoma and soft tissue impingement. Reduction was made manually with a point of reduction clamp and the fracture was stabilized with two parallel 0.062 k-wires (Figure 1(a)). A 3.4 mm drill, the Arthrex SwiveLock tap, the Arthrex no. 2 FiberTape, and Arthrex 4.75 mm SwiveLock anchor (Figure 1(b)) were obtained. The 3.4 mm drill was utilized for the anchor hole, being careful to maintain equidistance from the fracture site to the distal portion of the medial malleolus for the anchor hole (Figure 1(c)). A 4.75 mm SwiveLock tap was utilized down to the laser line (Figure 1(d)). The FiberTape was then wrapped around the distal k-wires and both ends were then placed through the 4.75 mm SwiveLock eyelet (Figure 1(e)). The FiberTape was then tightened to appropriate tension and the SwiveLock anchor was advanced burying the anchor past the black line with the Arthrex AO handle (Figure 1(f)). Prior to advancing the anchor, a gentle tap of the handle with the mallet was indicated for burying and alignment (Figure 1(g)). The FiberTape was then cut flush with the eyelet. After the fracture appeared to be in adequate anatomical alignment, the k-wires were bent, cut, and rotated to avoid soft tissue irritation (Figure 1(h)).

The incision was closed with 2-0 and 3-0 Monocryl for deep subcutaneous and subcuticular closure, respectively, and either nylon or skin staples for skin approximation. The patients were placed in a well-padded posterior. The patients were kept in the splint until suture removal and then transitioned to an immobilization boot. Weight bearing regimens were started 6–8 weeks postoperatively pending union and pain status. Physical therapy was utilized if patient weight bearing transition showed increased stiffness and tenderness.

The outcome measures include age, language, ethnicity, comorbidities, social history, extremity involved, fracture classification, results through economic costs (including initial product costs and return to operating room costs for irritation/revision/removal of hardware), and fixation results (including hardware status, healing status, pain status, and time to healing).

Specifically, ethnicity was recorded as Caucasian, Hispanic, or African American. Comorbidities (hypertension, diabetes, hyperlipidemia, etc.) were based on any medical conditions recorded in the chart other than their ankle fracture. Social history involved admitting to use of nicotine or alcohol or both. Extremity involved was the left lower extremity, the right lower extremity, or bilateral lower extremities. Preoperative images were classified as either isolated medial malleolar, bimalleolar, or trimalleolar fractures.

Economic cost comparison evaluated initial product cost (stainless steel wire, screw, k-wires, and Arthrex knotless tension band system) and return to operating room cost due to irritation/revision/removal of hardware at tension band site (KTB or WTB). It is important to note that cost for returning to the operating room was not taken directly from the patients recorded in this study. This data was obtained from every ankle fracture related hardware removal for the year of 2014 regardless of fixation used. Hardware removals for pilon or tibia plafond fixation were excluded.

Fixation results were determined and recorded by hardware status (hardware intact versus hardware removed), healing status (healed and consolidated > 12 weeks versus delayed union > 12 weeks) based on the clinical and radiographic guidelines of Corrales et al. [5] in Table 1, nonunion with the hardware intact, nonunion with the hardware removed, and time to healing. Time to healing duration was determined by radiographic and clinic chart reviews by the authors for union interval of the fracture site as well as evaluation to weight bearing status postoperatively. General nonspecific pain and pain noted at the tension band site associated with postoperative healing of the medial malleolus reduction site were all recorded.

The investigators collected the results and the data was analyzed with tables' generated using SAS software (9.3 version). Chi-square of independence test was used for bivariate analysis as well as logistic regression analysis. *p* values were reported with statistical significance defined at 5% (*p* < 0.05). These were evaluated and cross-analyzed for statistical inference as well as cost purposes.

## 3. Results

A total of 107 patients met our inclusion criteria. The WTB and KTB groups each had 89 and 18 patients, respectively. Descriptive variables of the patients that met the inclusion criteria are provided in Table 2. Not all of the numbers in the descriptive data equaled our total (*n* = 107) due to documentation variations in the chart review. The mean age of the patients was 46.2 ± 16.4 years for the WTB and 43.2 ± 11.1 years for the KTB at the time of surgery. The *p* value was 0.442 demonstrating no statistically significant age difference between groups.

**Figure 2:**
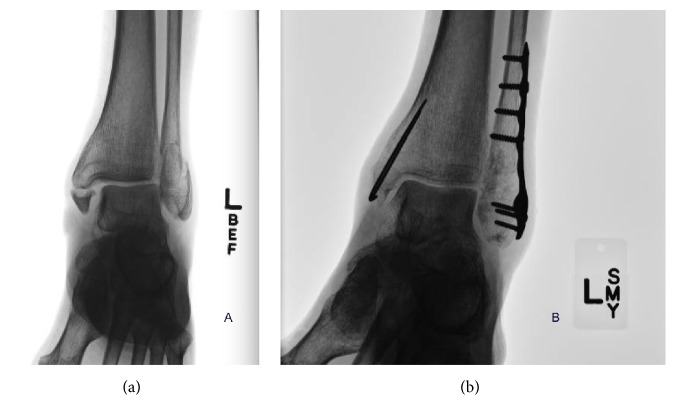
(a) demonstrates a preoperative bimalleolar fracture with a small medial malleolus contribution. (b) shows the mortise view postoperative fixation with the KTB.

Fracture classification and extremity involved are listed in Table 3. The majority of fractures were bimalleolar (57.0%) and both the right and left lower extremity were approximately equally involved (49.5% and 48.6%, resp.).  Figure 2(a) demonstrates a preoperative image of a bimalleolar fracture prior to definitive fixation being placed with the KTB and Figure 2(b) shows the mortise view of the KTB fixation.

**Figure 3:**
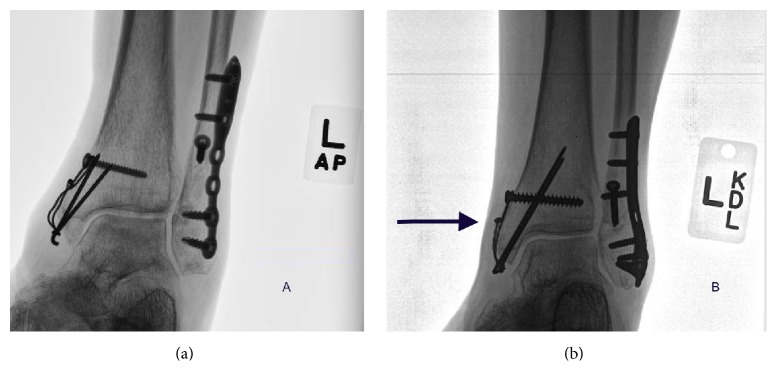
(a) demonstrates a common complication of stainless steel wire loosening for the WTB and subsequent irritation. (b) shows stainless steel wire irritation where the wire was cut after tensioning (arrow). Both patients went onto subsequent hardware removal.

Our analysis revealed that the initial product cost for the WTB is $44.25 (0.062 k-wires × 2, 3.5 cortical screws, and 18- or 22-gauge stainless steel wire) with *n* = 89 and the calculated total product cost for the group equaled $3,938.25. The hardware removal cost at our institution equals on average $8,288. Seven patients (7.9%) in the WTB required hardware removal due to irritation resulting in a $58,016.00 total cost for the group. The initial product cost for the KTB at our institution equals $616.56 (0.062 k-wires × 2, Arthrex no. 2 FiberTape, and SwiveLock with drill and tap) with *n* = 18 for a total product cost for the group of $11,098.08. None of the patients (0%) in the KTB required hardware removal and thus the total hardware removal cost was $0. These economic costs are summarized in Table 4 including the total product cost for each group, total hardware removal cost for the group due to irritation, and the total cost distributed per patient. The total cost distributed per patient represents the true cost of the operation ((product cost + hardware removal cost)/patient). The KTB group had a 13% lower true cost as compared to the WTB group. Figures 3(a) and 3(b) demonstrate the common complication of the WTB due to irritation and subsequent hardware removal.

Detailed results of fixation and pain outcome measures are listed in Table 5. Hardware status demonstrates total hardware intact for 100 (93.5%) patients, 82 (92.1%) for the WTB and 18 (100%) for the KTB. Hardware was removed in 7 patients (6.5%), 7 (7.9%) for the WTB and 0 (0%) for KTB (*p* = 0.9608). The rest of the data was equivocal for patients as demonstrated in Table 5. Pain measured generally and nonspecifically was significant at *p* = 0.0463.

## 4. Discussion

The previous KTB study by Clyde et al. [4] showed this technique to be biomechanically stronger, stiffer, and better pullout strength. They suggested that it could be clinically beneficial due to a lower profile, reduced discomfort at the medial malleolus site, and a lower risk of reoperation due to hardware irritation at the tension band site. The current study aims to evaluate and compare retrospective clinical outcome measures in regard to economic cost (initial product costs and return to operating room costs for hardware removal due to irritation) and fixation results (hardware status, delayed union, nonunion, time to healing, and pain) between the KTB and WTB procedures.

Limitations of our study include a small number of patients (*n* = 18) that met the inclusion criteria for the KTB. No significant age differences between the groups were found, however, strengthening group comparison. The no. 2 FiberTape is radiolucent and not visible on intraoperative imaging or postoperative radiographs and may be difficult to visualize loosening of the tension band construct or slippage on the bent 0.062 k-wire construct and can be considered a limitation. Moreover, even though the KTB did not experience any issues with k-wire loosening in the current study, there may be some migration in a longer follow-up period that could potentially require removal. We know that the KTB takes significantly less time to perform compared to the WTB; but given the data that was available and analyzed, we did not have enough information to quantify and prove the significance of the operative time. Partly, adding to our difficulty in quantifying, this was the variation in fractures (isolated medial malleolus, bimalleolar, and trimalleolar). In our analysis, we have therefore made the assumption that the initial fixation took the same time for both groups (KTB versus WTB) and have thus only used product cost for the initial surgery. Due to the WTB having a 7.9% hardware removal from irritation compared to 0.0% for the KTB, there is a 13% decrease in total cost for the KTB after the true cost was calculated (see Table 4). If we were able to also quantify the reduced operative time for the KTB procedure, we believe the economic advantage of the KTB construct would be further amplified. The economic data collected is based on the product cost at our institution, which may be different and varied at other facilities. We did experience one hardware removal for the KTB. This patient did not however meet our inclusion criteria. The patient was grossly noncompliant (drug abuse, severe alcohol abuse, and weight bearing on the operative extremity directly after fixation) and subsequent hardware removal was performed 10 days after the initial surgery due to postoperative infection and therefore was excluded from the study. The patient went onto developing a nonunion of the medial malleolus after continued noncompliance and had a septic ankle fusion at a later date. The majority of the variables for the fixation results were not significant and of equal value.

The *p* value for pain measured generally (Table 5) was significant at 0.0463 with the odds ratio calculated at 2.89 for this variable. This demonstrates that there are more likely to be general nonspecific complaints in the KTB group rather than the WTB group. Although these results are showing that there is significance with general pain, this is a subjective complaint and difficult to differentiate retrospectively. The pain measured at the KTB site after the incision healed and related to postoperative healing was not significant which dilutes the results of pain measured generally. Lastly, there were four total patients that were lost to follow-up; however, this did not affect the results as all had subsequent union 3 (2.8%) with hardware intact and 1 (0.9%) with hardware removed (see Table 5).

The literature is vast in reporting options for the repair of medial malleolar fracture types with suggestions of bone size, bone healing, compliance, strength, and hardware irritation, all being decisive factors for definitive fixation [2, 6–9]. The techniques range from hook plating, 3.5 mm bicortical screws for patients with concerning bone quality and standard 4.0 mm cancellous or cannulated screws for larger fragment sizes for medial malleolus fractures. The tension band wiring with a stainless steel system has been documented as offering superior strength and stiffness compared to screw fixation with a common goal of healing the fracture in a timely manner without associated risks [2, 6–9]. Even with superior results for medial malleolus fixation, Fowler et al. relate that the thin overlying skin, the anatomical location, and the soft tissue irritation appear to be reasons to avoid this approach [10]. This continued complication has evolved protocols directed towards low-profile tension band using braided suture to eliminate the irritation and the need for reoperation [10]. Despite this innovative procedure, the design showed inferior strength and stiffness in comparison to the traditional stainless steel wire tension band technique [10]. In contrast, Wright et al. [11] demonstrated superior strength and stiffness over the traditional stainless steel tension band technique when locking the FiberWire knot under greater tension. This bench-top study for transverse patella fracture fixation argues similar advantages of our study for less irritation and decreased risk of hardware removal when fixed with FiberWire [11]. Chen et al. [12] demonstrated a successful outcome for patella fractures fixed with a biodegradable tension band compared to tension band with metallic stainless steel wire in a randomized study. Their results demonstrated no clinical or radiographic differences between the two methods [12]. They also demonstrate that the strength of the biodegradable product was higher than the forces distributed on the patella without refracture or displacement [12]. Like this study [12], we found that the KTB can be successfully used to treat such fractures without the need for additional operations to remove the implant after union. Though Chen et al. did not have complications with reaction, biodegradable materials can produce an acute inflammatory soft tissue reaction that may resemble an infection or dehiscence. No cases have been published on this phenomenon with the Arthrex (Naples, FL) no. 2 FiberTape for its original design for lateral ankle instability augmentation with the* InternalBrace™*, Achilles detach/reattach procedure with the* SpeedBridge™*, or other studies that utilize this type of product. Loveday et al. [13] offer a technique based level 5 study utilizing a titanium suture anchor TWINFIX Ti 5.0 mm with braided suture no. 2 ULTRABRAID Smith & Nephew (Andover, MA). This study has a similar idea to the Arthrex (Naples, FL) KTB with the idea that is a simplified approach and would offer less irritation. The authors however discuss the technique without any supporting data [13] that we can compare with our findings. Patel et al. [14] offer a sled type technique with 2 prongs distally, a “U” shaped construct with two screws, and washers proximally for added stability. The authors demonstrated that their prongs are stronger and bigger than k-wire fixation and will prevent rotation. Additionally, they relate that because the screws are proximal they will prevent shear motion [14]. They argue that their technique avoids hardware pain and irritation over the standard WTB technique. Their results are inferior to the lag screw with less pullout strength and gapping noted anteriorly when tested. Unfortunately, a comparison study was not made with the traditional stainless steel WTB technique. The added hardware and bulk of the sled could cause irritation and difficulty in hardware removal should this complication arise. The results of the above literature innovated the Clyde et al. [4] study comparing the bench-top design of the KTB versus the traditional stainless steel WTB to prompt a construct with less irritation. With favorable mechanical outcomes (7.7% stronger, 33.2% stiffer, and 36.7% greater force for 2 mm displacement) for the KTB construct [4], an in vivo comparison for indications for clinical use of KTB system was appropriate. In 2010, Macario et al. [15] related that operating room costs were approximately $62/min. Our data of a 13% decrease in true cost reflects this report and justifies using the knotless tension band initially to avoid unnecessary returns to the operating room.

## 5. Conclusion/Summary

Previous restrictions have been discussed with only Sawbones® tibial model constructs being utilized for review of strength and stiffness with the KTB system in comparison to the traditional stainless steel WTB [4]. We offer successful in vivo cases of the knotless tension band (KTB) on medial malleolus fractures with the KTB technique. Even though there is equivocal data on the fixation results, the KTB total cost as calculated is 13% less than the WTB and this does not include the timing of the procedure. In our experience, although we could not quantify this in our current retrospective study, the time of the KTB procedure is less, further favoring KTB over WTB. We demonstrate that the KTB is comparable to the WTB while offering a hassle-free and irritation-free reduction. Further prospective studies are needed to measure KTB operating time of the procedure, direct patient costs, a validated scoring system, and patient satisfaction without additional frustration of hardware removal due to irritation.

## Figures and Tables

**Table 1:** It demonstrates the Corrales et al. [5] details of the synergistic effects of radiographic and clinical healing.

Clinical criteria to define fracture healing	Radiographic criteria to define fracture healing
(1) No pain or tenderness to palpation on physical examination	(1) Bridging of fracture with callus or trabecular bone
(2) No pain noted over hardware	(2) Bridging of fracture with two cortices
(3) No pain or tenderness with weight bearing	(3) Absence of hardware failure or loosening
(4) Ability to walk and perform activities of daily living without pain or tenderness	

**Table 2:** Descriptive outcomes of the patient population studied based on what was available in the medical record. Mean age is reported ± the standard deviation. All other outcomes are reported as the number of patients (and as a percentage of the group total).

Fixation	WTB	KTB
*Age*	46.45 ± 14.96	41.5 ± 18.25
*Primary language*		
English	68 (76.40%)	16 (84.21%)
Spanish	21 (23.59%)	3 (15.79%)
*Ethnicity*		
Caucasian	25 (39.05%)	8 (44.44%)
African American	7 (10.94%)	3 (16.67%)
Hispanic	32 (50%)	7 (38.89%)
*Comorbidities*		
Yes	47 (62.67%)	4 (25%)
No	28 (37.33%)	12 (75%)
*Nicotine abuse*		
Yes	47 (62.67%)	4 (25%)
No	57 (72.15%)	9 (52.94%)
*Alcohol abuse*		
Yes	20 (25.32%)	5 (29.41%)
No	59 (74.68%)	12 (70.59%)

**Table 3:** Fracture classification and extremity involved.

*Fracture classification*	
Total patients	107
Bimalleolar fracture	61 (57.0%)
Trimalleolar fracture	38 (35.5%)
Medial malleolus fracture	8 (7.5%)
*Extremity involved*	
Left lower extremity	52 (48.6%)
Right lower extremity	53 (49.5%)
Bilateral lower extremity	1 (0.9%)

**Table 4:** Details concerning economic costs based on data from our institution.

Economic cost	WTB	KTB
Total product cost for group (A)	$3,939.25	$11,098.08
Total hardware removal cost for group (B)	$58,016.00	$0
Total cost distributed per patient (A + B)/*n*	$696.12	$616.56
Hardware removal due to irritation	7.9%	0.0%

*13% lower true cost for KTB*		

**Table 5:** Fixation findings with *p* value as determined using a chi-square of independence test for bivariate analysis as well as logistic regression analyses.

Fixation results	Total fixation (WTB + KTB)	WTB *n* = 89	KTB *n* = 18	*p* value
*Hardware*				
Hardware intact	100 (93.5%)	82 (92.1%)	18 (100%)	0.9608
Hardware removed	7 (6.5%)	7 (7.9%)	0 (0%)	
*Healed hardware intact*				
Yes (consolidated)	80 (74.8%)	65 (73.0%)	15 (83.3%)	0.3645
No (delayed healing > 12 weeks)	27 (25.2%)	24 (27.0%)	3 (16.7%)	
*Healed hardware removed*	7 (6.5%)	7 (7.9%)	0 (0%)	0.9608
*Nonunion hardware intact*				
Yes	20 (18.7%)	17 (19.1%)	3 (16.7%)	0.8092
No	87 (81.3%)	72 (80.9%)	15 (83.3%)	
*Nonunion hardware removed*	0 (0%)	0 (0%)	0 (0%)	—
*Time to healing*				
Days	60.5 ± 32.1	59.5 ± 33.3	65.6 ± 25.6	0.6365
*Pain—general*				
Yes	42 (39.6%)	31 (34.8%)	11 (61.1%)	0.0463
No	64 (59.8%)	57 (64.0%)	7 (38.9%)	
*Pain—tension band site*				
Yes	27 (25.2%)	22 (24.7%)	5 (27.8%)	0.8054
No	79 (73.8%)	66 (74.2%)	13 (72.2%)	
*Lost to follow-up*				
Yes—healed hardware intact	3 (2.8%)	2 (2.2%)	1 (5.6%)	0.6592
Yes—healed hardware removed	1 (0.9%)	1 (1.1%)	0 (0%)	
No	103 (96.3%)	86 (96.6%)	17 (94.4%)	
